# Influencing the Conversation About Masculinity and Suicide: Evaluation of the Man Up Multimedia Campaign Using Twitter Data

**DOI:** 10.2196/mental.9120

**Published:** 2018-02-15

**Authors:** Marisa Schlichthorst, Kylie King, Jackie Turnure, Suku Sukunesan, Andrea Phelps, Jane Pirkis

**Affiliations:** ^1^ Centre for Mental Health Melbourne School of Population and Global Health The University of Melbourne Parkville Australia; ^2^ Heiress Films Sydney Australia; ^3^ Department of Business, Technology and Entrepreneurship Faculty of Business and Law Swinburne University of Technology Melbourne Australia; ^4^ Phoenix Australia Centre for Posttraumatic Mental Health The University of Melbourne Parkville Australia

**Keywords:** mental health, suicide, masculinity, men’s health

## Abstract

**Background:**

It has been suggested that some dominant aspects of traditional masculinity are contributing to the high suicide rates among Australian men. We developed a three-episode documentary called *Man Up*, which explores the complex relationship between masculinity and suicide and encourages men to question socially imposed rules about what it means to be a man and asks them to open up, express difficult emotions, and seek help if and when needed. We ran a three-phase social media campaign alongside the documentary using 5 channels (Twitter, Facebook, Instagram, YouTube, and Tumblr).

**Objective:**

This study aimed to examine the extent to which the *Man Up* Twitter campaign influenced the social media conversation about masculinity and suicide.

**Methods:**

We used Twitter insights data to assess the reach of and engagement with the campaign (using metrics on followers, likes, retweets, and impressions) and to determine the highest and lowest performing tweets in the campaign (using an aggregated performance measure of *reactions*). We used original content tweets to determine whether the campaign increased the volume of relevant Twitter conversations (aggregating the number of tweets for selected campaign hashtags over time), and we used a subset of these data to gain insight into the main content themes with respect to audience engagement.

**Results:**

The campaign generated a strong following that was engaged with the content of the campaign; over its whole duration, the campaign earned approximately 5000 likes and 2500 retweets and gained around 1,022,000 impressions. The highest performing tweets posted by the host included video footage and occurred during the most active period of the campaign (around the screening of the documentary). The volume of conversations in relation to commonly used hashtags (*#MANUP*, *#ABCMANUP, #LISTENUP,* and *#SPEAKUP*) grew in direct relation to the campaign activities, achieving strongest growth during the 3 weeks when the documentary was aired. Strongest engagement was found with content related to help-seeking, masculinity, and expressing emotions. A number of followers tweeted personal stories that revealed overwhelmingly positive perceptions of the content of the documentary and strongly endorsed its messages.

**Conclusions:**

The *Man Up* Twitter campaign triggered conversations about masculinity and suicide that otherwise may not have happened. For some, this may have been game-changing in terms of shifting attitudes toward expressing emotions and reaching out to others for help. The campaign was particularly effective in disseminating information and promoting conversations in real time, an advantage that it had over more traditional health promotion campaigns. This sort of approach could well be adapted to other areas of mental (and physical) health promotion campaigns to increase their reach and effectiveness.

## Introduction

### Background

In Australia, suicide is the leading cause of death in males aged 15 to 45 years [1]. Suicide rates of Australian men are three times higher than those of women [1], and this gender inequality is reflected internationally [2].

A number of factors have been found to contribute to higher suicide rates in men. Men are known to choose more lethal methods [3], show increased alcohol and substance use [4], and have less well-established coping strategies and social support networks [5-7]. In addition, men tend to avoid or delay help-seeking, particularly for emotional issues [8-10], have greater difficulties in recognizing negative emotions or distress [11], and are less aware of help services available to them [12,13]. Mostly, these factors are considered in isolation, with little regard to the mechanisms or driving forces that may underpin them [14].

It has been suggested that masculinity—or the rules prescribed by society about how men should live their lives [15]—help to explain gender disparities in suicide. Although gender roles have arguably changed and continue to vary with time and place, dominant masculine norms still exist in many western societies and influence how men navigate life. Independence, self-reliance, invulnerability, and the avoidance of negative emotions are some commonly expected behaviors [16], and these have been linked to men’s lower likelihood of seeking help or dealing with emotional problems [8,17,18]. Men often see help-seeking as a measure of weakness or failure and prefer solving problems on their own [10]. This stoic behavior can be lethal; self-reliance has been found to reduce and delay help-seeking and increase the likelihood of suicidal thoughts [14,19-22].

Seeking to change the picture on male suicide may benefit from challenges to some of these widely accepted male stereotypes. Discussing dominant masculinity and creating opportunities for redefining help-seeking strategies for men and opening up options for negotiating difficult life events can potentially have significant impacts. However, changing social norms is no easy feat, and requires holistic population-based interventions that are able to reach and engage with the wider community of men from all walks of life, backgrounds, and geographic locations. Developing and testing such interventions have the potential to take the field of suicide prevention forward; at present, only a relatively small number of population-based interventions have been shown to be effective (eg, restricting access to means and school-based awareness campaigns) [8].

### The Study

We developed one such intervention, with funding from the Movember Foundation. We collaborated with Heiress Films to create a three-episode television documentary called *Man Up*, seeking guidance from an advisory committee comprising representatives from various community organizations and other academic experts in men’s health. *Man Up* follows Sydney Triple M Radio personality, Gus Worland, across Australia as he explores the complex relationship between masculinity and male suicide. Gus meets a multitude of men who have struggled with suicidal thoughts or attempted suicide, as well as many individuals and organizations that are addressing the problem of male suicide by encouraging men to reach out to others. Gus is so affected by this that he creates a community service announcement (CSA) with the tagline “Man Up. Speak Up,” which serves as a call to action.

From the beginning, *Man Up* was conceived as something far greater than a television show. It was viewed as part of a multimedia intervention that also included components that took full advantage of the Web environment to kick-start a national conversation. Our collaborator, Heiress Films, created a website that acted as a hub for content and resources, housed the show’s trailer, and ran a 14-week social media campaign around the show. The campaign and related assets were released via 5 Web-based platforms (Twitter, Facebook, Instagram, YouTube, and Tumblr) over three phases. Phase 1 ran from August 15 until October 10, 2016, stopping just before the documentary was screened by the Australian Broadcasting Corporation (ABC). This phase encouraged men (and women) to watch the show. Phase 2 ran from October 11 to 30, 2016, coinciding with the 3 weeks over which the show was aired. This phase encouraged viewers to talk and share. Phase 3 ran from October 31, 2016 (the end of the screening period) to November 20, 2016, and prompted the community to take action. These phases corresponded to the campaign goals of creating a social media audience, promoting a conversation about masculinity and male suicide, and generating and maintaining engagement with the documentary and its content throughout the campaign and beyond. The campaign capitalized on our relationships with partner organizations, including Movember, beyondblue, Lifeline, Mindframe, Triple M Radio, and the ABC network. It also linked to events and trending topics (eg, the month of Movember, Mental Health Week, Father’s Day, and World Kindness Day).

The social media campaign was a crucial component of the overall intervention. There is increasing recognition that social media may have potential in suicide prevention, and may be particularly useful for otherwise hard-to-reach groups such as men [23,24]. Other social media interventions have been deployed in suicide prevention (eg, apps designed to support individuals at imminent risk and machine learning algorithms that aim to detect suicidal content or sentiment in web conversations) [25,26]. To our knowledge, however, there are no precedents for the way in which we used the social media campaign in our intervention.

This study focuses on the Twitter activity that was generated by the *Man Up* Twitter account (*manuptvseries*), evaluating the extent to which the campaign influenced the social media conversation about masculinity and suicide. It forms part of a larger evaluation of *Man Up*, the findings of which have been [27] or will be reported elsewhere (personal communication with M Schlichthorst, unpublished data, 2017; and K King, unpublished data, 2017).

The study addresses the following evaluation questions:

- What was the overall reach of the campaign and how did the audience engage with it?
- What were the highest and lowest performing tweets and what assets were associated with them?
- Did the Man Up campaign increase the volume of relevant Twitter conversations and, if so, was the increase sustained after the show?
- What were the main content themes with regard to audience engagement?

## Methods

### Overarching Approach

We used Twitter data to answer our evaluation questions. These data are easy to access and represent a real-time response, making them ideal for monitoring responses to events, patterns of communication, and general attitudes [28]. Twitter data have been used in studies on the mental health and suicide prevention field to understand how users discuss mental health issues and why they use social media to do so, to monitor attitudes toward depression and schizophrenia, to gauge how Twitter is used in the provision of feedback and support by mental health services, and to track suicide risk factors [29-33]. In the general health arena, Twitter data have also been used to monitor the impact of campaigns and related interventions (eg, in cervical cancer screening and smoking cessation) [34-36].

### Data Collection

We collected Twitter data from two sources, one via the social media tool *Twitter insights* and the other through harvesting original content tweets. In combination, these different sources provided us with the information we needed to address our 4 evaluation questions.

#### Twitter Insights Data

During the campaign, we downloaded weekly data reports from *Twitter insights* into an Excel file to monitor the growth and reach of the campaign, audience engagement with its content, and selected demographic variables such as age. These reports covered the full period of the campaign (August 15 to November 20, 2016) and enabled us to look at the campaign’s performance across its three phases. Specifically, we looked at “reactions” (retweets, replies, likes, profile clicks, URL clicks, hashtag clicks, expanded click, follows, and views) to tweets posted by *manuptvseries* between August 15 and November 20, 2016.

#### Original Content Tweets

We harvested original content tweets from a broader period to capture activity in the 14 weeks before the campaign (May 9 to August 14, 2016), the 14 weeks during the campaign (August 15 to November 20, 2016), and the 14 weeks after the campaign (November 21, 2016 to February 26, 2017). Tweets were harvested using the free-of-charge Twitter application programming interface and were included in the dataset if they used the hashtag #MANUP, which was the main hashtag used in promoting the campaign. These data included the full text of each tweet and additional information on when (eg, time and date) and by whom (eg, host, organization, private person, and public person or forum) it was tweeted. Data were stored in an external holding database by a US company called Rackspace. We had access to the data and could download customized datasets throughout the entire observation period (pre, during, and post campaign). A final dataset was imported into Excel, and a subset of that dataset was then imported into the qualitative data analysis software NVIVO Pro V11 developed by QSR International.

**Figure 1:**
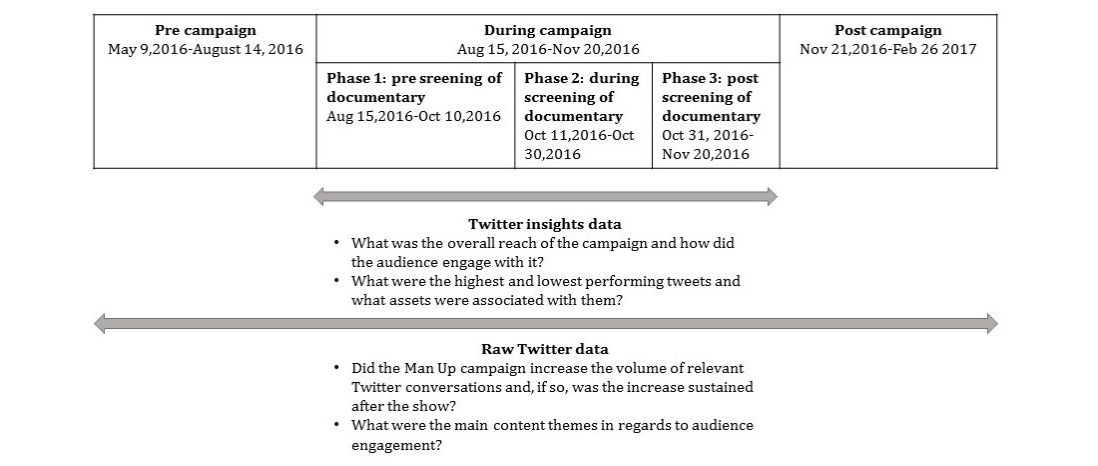
Data sources by time frames and evaluation questions covered.

Figure 1 summarizes the time periods covered by the two data sources and the evaluation questions each of them addressed.

### Data Analysis

All quantitative analyses were undertaken in Excel, and all qualitative analyses were performed in NVIVO Pro V11.

For evaluation question 1, we assessed the reach of and engagement with the campaign by using metrics from the Twitter insights data on followers, new followers, likes, retweets, and impressions (the number of people who saw campaign tweets on their timeline). We calculated frequencies, averages, and percentages for each as relevant, doing so for each of the three phases of the campaign.

For evaluation question 2, we determined the highest and lowest performing tweets in the campaign by ordering all tweets posted by *manuptvseries* based on an overall performance measure calculated as the number of “reactions” to these tweets, using Twitter insights data to do so. As each standard engagement measure assesses a different objective, we felt that an aggregate measure was a more democratic approach for comparing performance of tweets rather than using any single measure on its own. We took the “top 20” and “bottom 20” tweets, analyzed their content, and compared them in terms of their use of different assets.

For evaluation question 3, we used the original content tweets to determine whether the campaign increased the volume of relevant Twitter conversations, aggregating the number of tweets for selected campaign hashtags (*#MANUP, #ABCMANUP, #LISTENUP,* and *#SPEAKUP*) by week and plotting these for the precampaign period, the period of the campaign, and the postcampaign period. Specifically, we looked at the performance of *#MANUP* (occurring with or without other hashtags) and *#ABCMANUP, #LISTENUP,* and *#SPEAKUP* (occurring in combination with *#MANUP*). 

We calculated the average number of tweets per period and used *t* tests to test for significant differences in tweet volumes between the campaign period and the pre- and postcampaign periods. For evaluation question 4, we created a subset of data from the original content tweets to gain insight into the main content themes with respect to audience engagement with the campaign. We took all tweets that included the hashtag *#MANUP* and at least one other hashtag that had been used at least 10 times during the campaign by the *manuptvseries*. We then concentrated on those tweets in this group that were campaign-related (ie, tweets by the host, retweets of tweets by the host, or tweets that featured *Man Up* campaign content). We performed a thematic analysis of these tweets; MS read through 50% of the selected tweets and developed a preliminary coding framework with a list of themes. MS and KK then tested this framework using 10% of the tweets, revised it, and then retested it on another 10% of the tweets. MS and KK then finalized the framework by consensus (consulting with one another or another member of the team to resolve any disagreement), and each coded 50% of the total set of tweets.

## Results

### What Was the Overall Reach of the Campaign and How Much Did the Audience Engage With It?

Table 1 shows the reach of and engagement with the campaign. During the campaign, the number of followers of *manuptvseries* rose from 0 on August 15, 2016 to 1453 by November 20, 2016. The strongest growth in followers occurred during the time the documentary was screened (October 11 to 30, 2016). During this time, the campaign was most active with an average of 5 tweets per day being posted from the *Man Up* account. The number of likes and retweets was highest during this period. Impressions were strong before the show went to air as well as during the screening period. Over its whole duration, the campaign earned approximately 5000 likes and 2500 retweets and gained around 1,022,000 impressions. The beginning of the campaign saw more males being attracted to the campaign, but as time went by, there was a shift toward a more even distribution of genders among followers.

**Table 1:** Reach of and engagement with the *Man Up* campaign. Data source: Twitter insights; downloaded by Jackie Turnure.

Indicator		Prescreening of documentary (Aug 15-Oct 10, 2016)	During screening of documentary (Oct 11-30, 2016)	Post screening of documentary (Oct 31-Nov 20, 2016)
Frequent followers, n		519	1355	1453
Frequent new followers, n		519	836	98
Frequent likes, n		1500	2500	851
Average likes,n		26	118	41
Frequent retweets, n		656	1300	417
Average retweets, n		12	64	20
Frequent impressions, n		423,000	436,000	163,000
**Gender, n (%)**				
	Male	379 (73.0)	813 (60.0)	857 (59.0)
	Female	140 (27.0)	542 (40.0)	596 (41.0)

### What Were the Highest and Lowest Performing Tweets and What Assets Were Associated With Them?

Table 2 shows the type of assets associated with the highest and lowest performing tweets, as measured by “reactions.” A total of 12 (60%) of the highest performing tweets included a video, whereas only a single tweet (5%) among the lowest performing tweets did so. Furthermore, 18 (90%) of the lowest performing tweets included a link to an external source. Overall, the most successful tweet based on “reactions” was one that heralded the final episode before it went to air and provided a preview of the CSA that Gus created. The next most successful tweet promoted a relaunch of the trailer for the complete series. These videos were some of the main promotional assets for the show and were not only released via tweets but also across other Web-based platforms. The 20 highest performing tweets were all posted between October 11 and November 17, 2016, whereas the majority of the 20 lowest performing tweets (60%) were tweeted in August and September 2016, the first phase of the campaign.

### Did the Man Up Campaign Increase the Volume of Relevant Twitter Conversations and, if so, Was the Increase Sustained After the Show?

The original content tweets included 46,130 tweets that used the hashtag *#MANUP* (13,804 posted between May 9 and August 14 2016, pre campaign; 19,845 posted between August 15 and November 20, 2016, during the campaign; and 12,481 posted between November 21, 2016 and February 26, 2017, post campaign).

**Figure 2:**
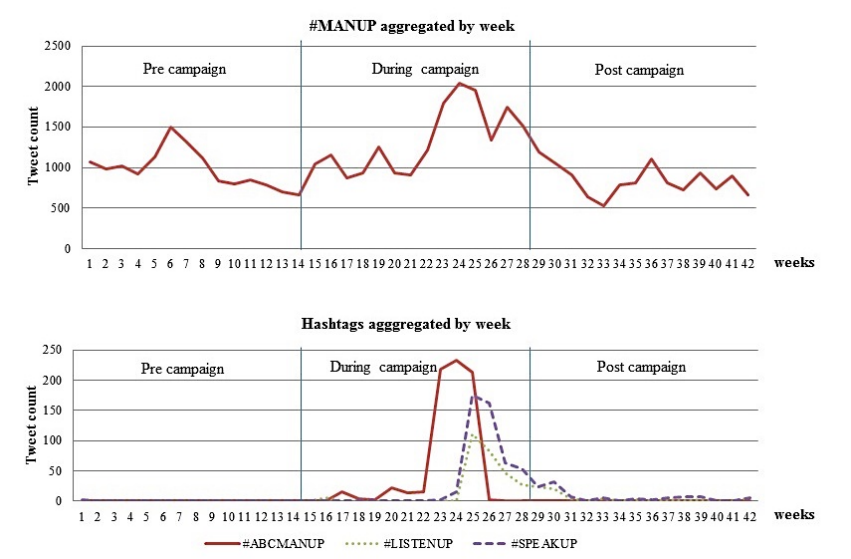
Hashtag frequencies on Twitter before, during, and after the campaign based on original content tweets.

Figure 2 shows the aggregated number of times *#MANUP* was tweeted (with or without other hashtags) in the three periods: pre, during, and post campaign. *#MANUP* was used an average of 979 times per week pre campaign. This rose to 1338 times per week during the campaign, and then dropped to 844 times per week post campaign. There was no significant difference in the use of *#MANUP* between pre and post campaign (*t*_1.683_, *P*=.052), but its use was significantly higher during the campaign period than either the pre (*t*_2.68_, *P*=.004) or post (*t*_4.13_, *P*<.001) campaign periods. Most of the increase was observed from week 22 (which corresponded with the airing of the documentary), and the use of *#MANUP* stayed at higher than average levels until week 27 (the end of the campaign).

Because *#MANUP* was commonly used in contexts that were unrelated to the campaign, we thought it would be useful to consider the performance of three other hashtags that were newly introduced by *manuptvseries* (*#ABCMANUP, #LISTENUP,* and *#SPEAKUP*), looking at them when they were used in combination with *#MANUP*. Figure 2 also shows these results. *#ABCMANUP* was used to promote the documentary on the ABC. This hashtag was introduced during the campaign and intensely used and shared in the period in which the documentary was aired (from week 22 to week 26), and then, its use was largely discontinued. *#LISTENUP* and *#SPEAKUP* were introduced by *manuptvseries* in the lead-up to the final episode in the context of the CSA. Again, their use peaked at this time but dropped as the campaign faded out.

**Table 2:** Engagement with tweets by asset type for the 20 highest and lowest performing tweets. Data source: Twitter insights.

Asset type		Frequency, n (%)	Sum of reactions^a^	Sum of retweets	Sum of replies	Sum of likes	Sum of media views
**Top 20 tweets**							
	Video	12 (60)	14,069	240	28	395	12,856
	Graphic	5 (25)	1599	148	26	293	542
	Link	2 (10)	2205	43	3	62	2014
	GIF^b^	1 (5)	897	2	0	6	872
	Total	20 (100)	18,770	433	57	756	16,284
**Bottom 20 tweets**							
	Link	18 (90)	62	2	0	42	0
	Graphic	1 (5)	3	0	0	2	1
	Video	1 (5)	3	0	0	1	0
	Total	20 (100)	68	2	0	45	1

^a^Aggregate of all engagement including video views.

^b^GIF: Graphics Interchange Format .

### What Were the Main Content Themes With Regard to Audience Engagement?

We identified a subset of 2093 tweets that included the hashtag *#MANUP* and at least one other hashtag that had been used at least 10 times during the campaign by *manuptvseries*. Of these, 1876 were campaign-related tweets, and we focus on these here. Of the campaign-related tweets, 229 were from *manuptvseries*. In total, 417 (22.2%) of the campaign-related tweets were original tweets and 1459 (77.77%) were retweets. The majority (1328 or 70.79%) were neutral in tone and did not show any specific expression of sentiment. In addition, 544 (29.0%) tweets provided positive feedback about the campaign or endorsed it, and 4 (<1%) took a negative stance or criticized the content of the campaign. The 3 tweets listed below provide examples of positive, neutral, and negative sentiment, respectively:

RT @ManUpTVSeries: Great to see the conversation getting started. #ManUp #SmashTheStigma #itsokaytotalk

@newlz in @HuffPostAU Talking. Listening. Sharing. These are the tenets that now drive me. #ManUp #weneedtotalk

@username Except if you’re a male victim of #domesticviolence - then you get told to #ManUp and discriminated against #Reality #ABCManUp

Several content themes were identified in the tweets: expressing emotions, mental health issues with the subthemes of mental health and suicide, men’s issues with the subthemes of being a man and fathering and raising boys, help-seeking with the subthemes of providing options for help and other help-seeking, personal stories, and supporting others. Table 3 summarizes these content themes for the 1876 campaign-related tweets and indicates whether they were original tweets or retweets.

*Expressing emotions* was the theme identified most commonly, occurring in 710 tweets (37.8% of all campaign-related tweets). The strength of this theme is not surprising, as the CSA that was produced and released in the final episode of the show encouraged men to open up and express difficult emotions. The tweet that promoted the CSA achieved 125 retweets on its own and was the highest performing tweet of the entire campaign measured by the number of “reactions.” The number of the tweets related to expressing emotions highlighted the difficulties men experience in opening up and asking for help in difficult times, and the stigma around mental health. Some tweeters acknowledged that *Man Up* made them cry, or that they had opened up to someone after watching the show. Examples are provided here:

RT @ManUpTVSeries: The need for men to be emotionally honest is greater than ever. Interesting #blog post from @3DMathW. #ManUp

RT @username: Not ashamed to admit a few tears have been shed watching #ManUp over the last few weeks #ABCManUp

RT @ManUpTVSeries: The strong silent type might be sexy in films, but it’s unhealthy in real life. #ManUp #itsokaytotalk

**Table 3:** Content themes of the 1876 campaign-related tweets. Data source: Original content tweets.

Theme		Description	Tweet count n (%)	Retweet n (%)	Original tweet n (%)
Expressing emotions		Tweets that include topics such as speaking up, opening up, talking about uncomfortable issues, breaking down stigma, and crying	710 (100.0)	550 (77.5)	160 (22.5)
**Mental health issues**					
	Mental health	Tweets that indicate mental health, depression, or posttraumatic stress disorder, or include materials and links that discuss these topics, or use the hashtag mental health	410 (100.0)	300 (73.2)	110 (26.8)
	Suicide	Tweets that indicate suicide or suicide prevention, or include materials and links to sites that discuss these topics, or use the hashtag suicide	182 (100.0)	140 (76.9)	42 (23.1)
**Men’s issues**					
	Being a man	Tweets that discuss the concept of masculinity or challenge the concept of masculinity	165 (100.0)	125 (75.8)	40 (24.2)
	Fathering and raising boys	Tweets that encourage discussion about what it means to be a father and raising boys	82 (100)	63 (77)	19 (23)
**Help-seeking**					
	Providing options for help	Tweets that provide information on help services and encourage their use	165 (100.0)	147 (89.1)	18 (10.9)
	Help-seeking other	Tweets mentioning other content on help-seeking (ie, not about providing options for help)	96 (100)	79 (82)	17 (18)
	Personal stories	Tweets relating personal stories, written in the individual’s own voice, and revealing detail about the person (not commentaries or statements)	101 (100)	61 (60)	40 (40)
	Supporting others	Tweets about providing support to others, support options and general advice	78 (100)	63 (81)	15 (19)

The theme of *mental health issues and suicide* occurred in 484 tweets (25.8% of all campaign-related tweets). Moreover, 410 of these tweets featured references to *mental health issues* and 182 made mention of *suicide* (with 108 covering both). Tweets that exemplified this theme provided information on mental health issues or suicide, aimed to raise awareness about them, and encouraged people to speak up about them. There was some overlap in the two subthemes as some tweets referred to mental health more broadly and suicide more specifically within the one tweet. Examples of tweets involving the theme of *mental health issues and suicide* are provided below:

Massive shout out to @GusWorland. As sufferer of PTSD for 8 years as Ex-Cop #ManUp really hit home hard. Congrats mate. #ManUp #SpeakUp

RT @username: Depression is an illness people can help you recover from #ManUp on ABC at the moment is great. #mentalhealthweek

Suicide has touched so many lives, I’m tearing up already #ABCManUp #manup #blackdoginstitute #lifeline #beyondblue

RT @ManUpTVSeries: How suicide can become “contagious” to other at-risk young men. Important piece in @DailyMailAU. #ManUp #SpeakUp

*Men’s issues* were also a relatively common theme, accounting for 211 tweets (11.2% of all campaign-related tweets). In addition, 165 of these tweets related to *being a man* and 82 contained content about *fathering and raising boys* (with 36 making reference to both). Tweets that related to *being a man* encouraged discussions about the concept of masculinity, provoking and challenging stereotypical masculinity, and encouraging others to engage in a conversation about masculinity and what it means to be a man. Examples included:

Inspired by the #ManUp TV series, we have a chat about what it means to be a MAN https://t.co/0a6wqtimAP #Movember #mentalhealth #goodcause

RT @ManUpTVSeries: “Our ideals of #masculinity have shifted.” @MichaelGLFlood is one of our #RealAussieblokes. #ManUpâ€¦

RT @OliShawyer: This ad made me cry. I’m covered in Tatts. I ride a Harley. And I’m crying. Try tell me that’s weak. #manup #speakup thank you @gusworland

Tweets on *fathering and raising boys* provided information on these topics. These were often linked to notions of being a man and raised issues around the expectations placed on boys as they grow up. Some prompted consideration of what could be done differently in raising boys today to avoid reinforcing traditional stereotypes. Examples included:

RT @ManUpTVSeries: We need to have a hard look at how we raise our boys. #ManUp #raisingboys #ChildHealthDay @harkin_tom

RT @username: #ABCManUp #ManUp Let’s start helping boys from a young age. Dads need to give them cuddles, talk about feelings. #natural

RT @Top_Blokes: Providing boys with positive older male mentors is important to keep them safe and alive #ABCManUp #ManUp

The theme of *help-seeking* was evident in 202 tweets (10.8% of all campaign-related tweets). In addition, 165 of these tweets embodied the subtheme of *providing options for help* and 96 were classified as falling under the subtheme of *other help-seeking* (with 59 exemplifying both subthemes). Tweets that exemplified *providing options for help* promoted help-seeking in general and pointed to particular services more specifically (eg, Mindframe, Lifeline, headspace, SANE Australia, Kids Helpline, and MensLine). A tweet that typified this theme was:

RT @ManUpTVSeries: #RealTalk for a sec: if you or a mate are doing it tough please call @LifelineAust on 13 11 14 #ItsOkayToTalk #ManUp

The tweets in the theme *other help-seeking* mentioned two different aspects of help-seeking. They provided information on male help-seeking behavior and the issues that arise from it, thereby providing opportunity to create awareness and reflection on the issue. They also gave advice on taking action in help-seeking and motivating behavior change. Examples included:

RT @ManUpTVSeries: #ManUp survey: over 56% of men would rather manage themselves than seek professional help. #weneedtotalk #questmh

RT @ManUpTVSeries: Shame could be a big reason why…some men [don't] ask for help. Beautiful #blog from @drmwroberts #ManUp #NoShame

Another key theme— *personal stories* —was embodied in 101 tweets (5.4% of all campaign-related tweets) where people opened up to tell their story in their own voices. The stories included reflections on the experience these people had watching the documentary, and things that may have happened to them or someone they knew. Furthermore, they included responses to a collection of self-reflective portraits revealing personal struggles and hope that were released on the *Man Up* website in a segment called “Aussie Blokes.” Examples included:

Absolutely opened my eyes to the daily struggles of both genders. #ManUp I gave my fiancÃ© a big hug after watching that tonight #ABCManUp

I’ve lost 3 mates to suicide. Wish I noticed what they were going through. Don’t #Manup, seek help cuz there’re many out there #ABCManUp.

When I was younger, everything I did was bulletproof #RealAussieBlokes #ManUp #exercise.

The final theme was *supporting others.* This was apparent in 78 tweets (4% of all campaign-related tweets). These tweets discussed the importance of supporting others and reaching out to those in need, and the skills of listening. Examples included:

Powerful stuff @ManUpTVSeries #ManUp #ABCManUp We have a way to go to support our young men on their journeys. It’s a tough world we live in.

Sometimes the most important thing is just to listen. @BeardedGenius in @JOE_co_uk. #ManUp #SpeakUp #ListenUp

Across all themes and subthemes, the majority of tweets were retweets rather than original tweets, indicating high levels of engagement. Proportionally, the highest percentage of retweets was for *providing options for help* (90% retweets; 10% original tweets), and the lowest percentage was for *personal stories* (60% retweets; 40% original tweets).

## Discussion

### The Success of the Man Up Twitter Campaign

We evaluated the extent to which the *Man Up* Twitter campaign influenced the conversation about masculinity and suicide among Australian men. The campaign was very successful in reaching an audience that was engaged with its content, as evidenced by the number of “reactions.” Not surprisingly, campaign performance was highest during the period in which the show was aired, but social media conversations continued and followers stayed engaged beyond this. In fact, social media channels are still active today.

Certain elements of the campaign were particularly successful. These included tweets relating to the CSA Gus created on screen that encouraged men and boys to reject the constraints of traditional masculinity and speak up if they were facing tough times, as well as tweets featuring the trailer and episode teaser videos. The conversations generated by the campaign aligned with its major themes of expressing emotions, mental health issues and suicide, being a man and fathering and raising boys, help-seeking, personal stories, and supporting others. Again, related to the CSA release, the most discussed theme was expressing emotions.

The large number of positive comments indicated great acceptance and endorsement of the documentary. Many tweets welcomed open discussion of masculinity and male suicide and embraced the call for men to open up and express their emotions. There was a sense that for some men, questions on male identity and masculine norms had been bubbling beneath the surface, and the campaign gave men permission to articulate these thoughts and emotions. For others, ideas around changing the way we look at masculinity and its link to suicide appeared to be new, thought-provoking, and even challenging. These differing perspectives added to the richness of the discussion.

### The Man Up Twitter Campaign as Part of a Strategic Multimedia Intervention

The Twitter campaign occurred as part of a strategic multimedia campaign. It was rolled out around the documentary via three phases, each of which aligned with a specific goal, and it was one component of the broader campaign. A significant proportion of the content released by *manuptvseries* was directly related to the documentary, as were many of the comments tweeted by the general public. We are confident that the Twitter campaign had an independent effect in terms of influencing the social media conversation about masculinity and suicide, but it is difficult to tease out its independent contribution to the overall success of the *Man Up* enterprise.

### Contributing to the Broader Field of Suicide Prevention

As noted earlier, there is still much that is unknown about what works and what does not work in suicide prevention. There are relatively few interventions for which there is indisputable evidence of effectiveness [37], although improvements are being made. The jury is still out on suicide prevention media campaigns [38], although there is emerging evidence that they may work for some audiences. Most of the media campaigns that have been evaluated have tended to be fairly traditional, typically involving brief CSAs that may be delivered through different channels. Few have targeted men specifically, although some have targeted groups (eg, police) in which men may be well represented. Our intervention had the luxury of being more extensive, partly because it was underpinned by a three-episode documentary and partly because it capitalized on the digital environment to get its message out. Harnessing the media in suicide prevention in a nontraditional manner certainly seems to show promise.

### Limitations

Both datasets that we used here had certain limitations, and these should be considered in interpreting our findings. In the case of the Twitter insights data, the key limitation relates to our measurement of success. We used the standard metrics of numbers of followers, likes, and retweets, and we created an aggregate measure, which we termed “reactions” (retweets, replies, likes, profile clicks, URL clicks, hashtag clicks, expanded click, follows, and views) to rank tweets in terms of their performance over the duration of the campaign. The way we aggregated “reactions” is open to challenge, although, as noted above, we felt that it was a democratic approach. In addition, the fact that we monitored tweets’ performance over the duration of the campaign disadvantaged tweets from earlier in the promotion cycle as these had less exposure because of lower numbers of followers and generally lower engagement with the campaign. This comparison could be improved by monitoring the performance of each tweet over the same duration (eg, for the first 2 weeks after it was posted) and creating some sort of performance per follower weighting, but “leveling the playing field” in this way was beyond the scope of our current endeavors.

In the case of the original content tweets, the main limitation relates to the way in which we were able to capture tweets relating to the campaign. We monitored the use of the hashtag *#MANUP*, assuming that this would provide a window into the effectiveness of the social media campaign. The difficulty with this approach was that *#MANUP* was already commonly used worldwide in different contexts (eg, politics, sports, and entertainment), often with negative connotations (ie, promoting messages such as “harden up” and “tough it out”). The volume of tweets that were unrelated to our campaign created a challenge for identifying the relevant content that would tell the story of our campaign. For this reason, we also looked at a subset of *#MANUP* paired with other campaign-related hashtags for more in-depth qualitative analysis.

There are also limitations associated with using Twitter data in general. These data present something of a skewed picture because they can only represent those who are active on Twitter. In Australia, only about 19% of Internet users use Twitter, and a majority of these are relatively young [39]. This means that our Twitter evaluation data will be likely to have some inherent biases.

### Conclusions

The *Man Up* Twitter campaign triggered conversations about masculinity and suicide that otherwise may not have happened. For some, this may have been game-changing in terms of shifting attitudes toward expressing emotions and reaching out to others for help. The campaign was particularly effective in disseminating information and promoting conversations in real time, an advantage that it had over more traditional health promotion campaigns. This sort of approach could well be adapted to other areas of mental (and physical) health promotion campaigns to increase their reach and effectiveness.

## Abbreviations

ABC: Australian Broadcasting Corporation
CSA: community service announcement
